# Effects of combined exercise training with or without nutritional counseling on cardiovascular risk factors in older adults with hypertension: a pragmatic randomized trial

**DOI:** 10.1038/s41371-026-01217-7

**Published:** 2026-09-03

**Authors:** Vinícius Mallmann Schneider, Laura de Lima Silva, Gabriel Carvalho, Gabriel Lemes, Dalva Muniz Pereira, Rodrigo Ferrari

**Affiliations:** 1 Postgraduate Program in Cardiology, School of Medicine, Universidade Federal do Rio Grande do Sul, Porto Alegre, RS 90035-903 Brazil; 2 Sports and Exercise Training Study Group, Clinical Research Center, Hospital de Clínicas de Porto Alegre, Porto Alegre, RS 90035-903 Brazil; 3 Postgraduate Program in Human Movement Sciences, School of Physical Education, Universidade Federal do Rio Grande do Sul, Porto Alegre, RS 90690-200 Brazil; 4 Present Address: Postgraduate Program in Cardiology, School of Medicine, Universidade Federal do Rio Grande do Sul, Porto Alegre, RS 90035-903 Brazil

**Keywords:** Risk factors, Clinical trials

## Abstract

High blood pressure and elevated glycemia increase cardiovascular risk in older adults. Physical activity and diet are cornerstone non-pharmacological strategies to manage these risk factors. However, interventions in community settings focusing on the combination of exercise and nutrition are scarce. This pragmatic randomized controlled trial compared combined exercise training with versus without nutritional counseling on fasting blood glucose (primary outcome), blood pressure, body composition, and cardiorespiratory fitness in older adults with hypertension. Thirty-two participants (50–80 years) were randomly assigned to exercise training alone (EX; *n* = 16), comprising walking/jogging and calisthenics (60 min per session, twice weekly), or exercise training plus nutritional counseling based on the Brazilian Dietary Guidelines (EX + N; *n* = 16; six biweekly group sessions emphasizing food quality and reduction of ultra-processed foods without energy restriction). The 12-week combined training was conducted in outdoor athletics track. Data were analyzed using generalized estimating equations with intention-to-treat principle. Between-group differences used Bonferroni correction, effect sizes used Hedges’ g, and significance was α < 0.05. Twenty-nine participants completed the intervention. Fasting blood glucose was lower in EX + N than EX (-10 mg/dL; *P* = 0.004; g = 1.07). Systolic blood pressure decreased within EX + N (−10 mmHg; *P* = 0.034; g = 0.89) but not within EX, with no between-group difference (*P* = 0.220; g = 0.43). No between-group differences for diastolic blood pressure, body composition, or cardiorespiratory fitness. Nutritional counseling added to a community-based exercise program reduced fasting blood glucose compared with exercise alone, supporting the addition of nutritional counseling to exercise as a pragmatic strategy.

## Introduction

High blood pressure and hyperglycemia are critical drivers of cardiovascular disease and mortality, especially among aging adults [1]. Their coexistence frequently amplifies cardiovascular risk beyond the independent effect of either condition [2]. The two conditions are mechanistically linked through shared pathways that drive vascular injury, including activation of the renin-angiotensin system, oxidative stress, endothelial dysfunction, and arterial stiffening [3]. These processes couple macrovascular and microvascular damage, accelerating target-organ injury when both conditions are present [4]. Regular exercise and diet are two excellent non-pharmacological strategies to simultaneously improve metabolic and cardiovascular parameters [5, 6]. Therefore, lifestyle strategies composed of nutrition and exercise are widely recommended to reduce cardiovascular risk [7].

Despite established efficacy, most combined nutrition and exercise interventions are implemented under controlled conditions that limit their external validity and real-world applicability [8, 9]. Delivering combined exercise and nutritional counseling in community settings rather than clinical or laboratory environments reduces barriers to access, leverages peer support, and increases the likelihood of sustained behavior change compared with traditional intensive interventions [10]. Exercise protocols relying on specialized equipment/facilities or rigid caloric prescriptions often hinder long-term adherence and scalability [11]. Walking and calisthenic exercises have demonstrated promising results in individuals with hypertension and diabetes [12, 13]. These low-cost exercise protocols can be performed at home, park, hospital, or any other place, facilitating better compliance to exercise. Dietary guidelines have prioritized qualitative patterns over isolated nutrient targets [14]. For instance, The Dietary Guidelines for the Brazilian Population emphasize food processing levels, home preparation, and meal planning, representing a low-cost, culturally adaptable approach [15]. However, whether integrating these pragmatic approaches yields clinically meaningful results in higher risk populations is unknown.

The purpose of this study was to compare combined exercise training alone or associated with nutritional counseling on cardiovascular risk factors in older adults with hypertension. The primary outcome was fasting blood glucose. Blood pressure, body composition, and cardiorespiratory fitness were secondary outcomes. We hypothesized that adding nutritional counseling to combined exercise training would produce a greater reduction in fasting blood glucose than exercise training alone.

## Materials and methods

### Study design

This 12-week, parallel-group, superiority, pragmatic randomized controlled trial was conducted within a community-based program at the Federal University of Rio Grande do Sul in Porto Alegre, Brazil. The participants were randomized to receive either combined training alone (EX) or combined training plus nutritional counseling (EX + N). This trial was designed and reported according to the CONSORT guidelines for pragmatic trials [16]. The degree of pragmatism of the trial was characterized using the PRECIS-2 tool [17], which rates nine design domains from 1 (very explanatory) to 5 (very pragmatic). The trial was predominantly pragmatic. Most domains were rated 3 or 5, reflecting recruitment from an existing community program, delivery in a community outdoor setting, a low-cost and adaptable intervention, minimal adherence enforcement, and an intention-to-treat analysis. The primary outcome and the follow-up assessments were more explanatory, reflecting the use of a physiological marker and standardized pre- and post-intervention measurements. The full domain scores and the PRECIS-2 wheel are presented in Supplementary Figure. This trial was not registered in a public clinical trials registry.

### Participants

Participants were 50–80 years old and had previous physician-diagnosed primary hypertension (1–4 antihypertensive medication use). The exclusion criteria were as follows: musculoskeletal limitations affecting participation in the exercise program; cardiovascular disease such as acute myocardial infarction, angina, or stroke within the past 24 months; heart failure classified as NYHA III or IV; chronic diseases requiring intensive management (e.g., cancer, dialysis-dependent kidney disease, multiple sclerosis, and Parkinson’s disease); body mass index ≥40 kg/m²; diabetes mellitus with target-organ damage; and inability or unwillingness to provide informed consent. They were recruited from an ongoing community-based exercise program and enrolled after a 3-month interruption in supervised training, so they were previously active but detrained at baseline. All participants received detailed information about the study procedures and provided written informed consent. The study was approved by the Research Ethics Committee of the Federal University of Rio Grande do Sul (UFRGS; No. 92345825.5.0000.5347) and was conducted in accordance with the principles of the Declaration of Helsinki.

### Procedures

The baseline assessment included two morning visits to the laboratory. During the first visit, a standardized interview and a comprehensive clinical examination were performed. Two independent evaluators who were blinded to study interventions conducted these assessments. Outcome assessments were conducted at baseline and after 12 weeks of intervention and included the following variables: office blood pressure, anthropometrics, and blood glucose.

The exercise training program was prescribed by qualified exercise professionals and nutritional counseling by registered dietitians. The interventions were carried out in outdoor athletics track and meeting room for exercise and nutrition, respectively. The interventions (EX and EX + N) lasted 12 weeks.

#### Combined aerobic and resistance training

The exercise sessions were performed two times per week, on Tuesdays and Thursdays (morning sessions: 7:30-09:30 a.m./ afternoon sessions: 5:00-7:00 p.m.). Participants performed both modalities in the same session, with resistance exercises immediately followed by aerobic exercise. Each session lasted 60 min, starting with 30 min of the following calisthenic exercises: push-ups, squats, inverted rows, calf raise, unilateral balance, and sit-ups (2-3 × 12–20 repetitions or 45 seconds for unilateral balance / 1-min interval between sets), followed by 30 min of aerobic exercise (walking or running). The intensity was monitored using rate of perceived exertion (Borg CR-10 scale) [18], with a target effort of 4–5 (somewhat hard, hard/intensity) for both exercise modalities. No progression was implemented during the 12-week protocol, and adherence was monitored by recording the attendance to the exercise sessions.

#### Nutritional counseling

The intervention included verbal guidance on healthy eating and was based on the recommendations of the Brazilian Dietary Guidelines [19], without any prescribed individualized diets or energy-restricted meal plans. The intervention content was structured according to the NOVA food classification system, which categorizes foods by degree of processing (unprocessed/minimally processed, culinary ingredients, processed, and ultra-processed) and their impacts on health and diet quality [20]. Participants of EX + N attended six biweekly group meetings (30 min per session). Each counseling session was delivered to groups of up to 8 participants, scheduled at two separate times to keep groups small. Sessions were conducted before the exercise program and covered the following topics: (week 1) current eating habits and differences between food processing categories; (week 3) food label reading and interpretation; (week 5) ultra-processed food consumption and sodium content; (week 7) ultra-processed food consumption focusing on sugar and fat content; (week 9) culinary skills as a strategy to promote health and food autonomy; and (week 11) promotion of healthy eating patterns and appreciation of regional foods. The approach during these meetings aimed to encourage critical understanding of food choices and support healthier eating behaviors.

#### Outcome assessments

The primary outcome was fasting blood glucose. Blood pressure, body composition, and cardiorespiratory fitness were predefined secondary outcomes.

#### Fasting glucose

levels were measured using a validated glucometer (Accu-Chek®; Roche Diagnostics, Germany) following standard capillary blood sampling procedures. The participants were instructed to fast for a minimum of 8 h and a maximum of 12 h before each assessment. Participants were instructed to maintain their usual dietary habits before both the baseline and post-intervention assessments, so that the measure reflected habitual intake rather than a short-term dietary change.

#### Office blood pressure

was measured after 10 min of seated rest using a validated automatic oscillometric device (HBP-1100; OMRON Healthcare, Japan). Participants were instructed to avoid physical exercise and alcohol consumption for 24 h before the assessment. Three consecutive measurements were obtained at 1-minute intervals on the arm presenting the highest initial systolic BP, and the mean of the last two readings was used for analysis. Pulse pressure (PP) was calculated as systolic BP minus diastolic BP, mean arterial pressure (MAP) as (systolic B*P* + 2 × diastolic BP) / 3, and double product (DP) as systolic BP multiplied by heart rate (HR).

#### Anthropometrics

Body weight was assessed using a digital scale, and height was measured using a stadiometer. Waist circumference was measured at the midpoint between the lowest rib and the iliac crest, with the participant standing at the end of normal expiration using a non-elastic anthropometric tape. Body mass index (BMI) was calculated as body mass divided by height squared (kg·m⁻²).

#### Functional capacity

was assessed through the Six-Minute Walk Test (6MWT) [21]. The test was performed indoors on a flat, firm-surfaced, 30-m corridor, with cones placed at each end to demarcate the turnaround points. Participants were instructed to walk as far as possible during the six-minute period. The total distance covered, in meters, was recorded as the test score. Maximal oxygen uptake (VO₂_max_) was estimated from 6MWT performance using the following predictive equation: VO₂_max_ (mL·kg⁻¹·min⁻¹) = 59.44 − (3.83 × sex) − (0.56 × age) − (0.48 × BMI) + (0.04 × 6MWT), where sex was coded as 1 for men and 2 for women, age was entered in years, BMI was calculated as body mass divided by height squared (kg·m⁻²), and 6MWT denotes the distance covered in meters [22].

### Sample size and randomization

A sample size of 32 participants provided adequate statistical power to detect a between-group difference of −8 mg/dL in the fasting blood glucose level, assuming a standard deviation of 8 mg/dL, a two-sided α of 0.05, and 80% power. This justification was based on parameters commonly reported in previous studies [23] and was calculated using the PSS Health online tool [24]. Randomization occurred at the individual level using a computer-generated sequence stratified by BMI with a fixed 1:1 allocation ratio. A researcher not involved in participant recruitment, intervention delivery, or outcome assessment generated and stored the randomization list. Allocation concealment was maintained by releasing group assignments only after all baseline assessments were completed, and the study staff responsible for enrollment had no access to the randomization list. Participants were informed of their assigned group only after baseline testing. Outcome assessors and data analysts remained blinded to group allocation throughout the study, whereas blinding of participants and exercise professionals was not feasible because of the nature of the intervention.

### Statistical analysis

Outcomes were analyzed using a complete analysis set that included all randomized participants in accordance with the intention-to-treat principle. The statistician was not involved in participant recruitment or allocation to intervention sessions and remained blinded to the group assignment throughout the analyses. The assumption of normality was evaluated using the Shapiro–Wilk test and visual inspection of the Q–Q plots. Baseline continuous variables were compared between groups using the independent sample Student’s t-test, whereas categorical variables were analyzed using the chi-square test. Primary and secondary outcomes were analyzed using generalized estimating equations (GEE), accounting for a repeated-measures design. The models included fixed effects for group (EX and EX + N), time (pre- and post-intervention), and group-by-time interaction. Post hoc comparisons were performed using estimated marginal means with Bonferroni adjustments for multiple comparisons. For the primary outcome (fasting blood glucose), the analyses were adjusted for baseline glucose values. All other outcomes were analyzed without adjusting for covariates. Effect sizes were calculated using Hedges’ g and interpreted as small (<0.50), medium (0.50–0.79), or large (≥0.80) [25]. Statistical significance was defined as a two-sided P-value of <0.05. All statistical analyses were performed using IBM SPSS Statistics for Windows (version 25.0; IBM Corp., Armonk, NY, USA).

## Results

**Fig. 1:**
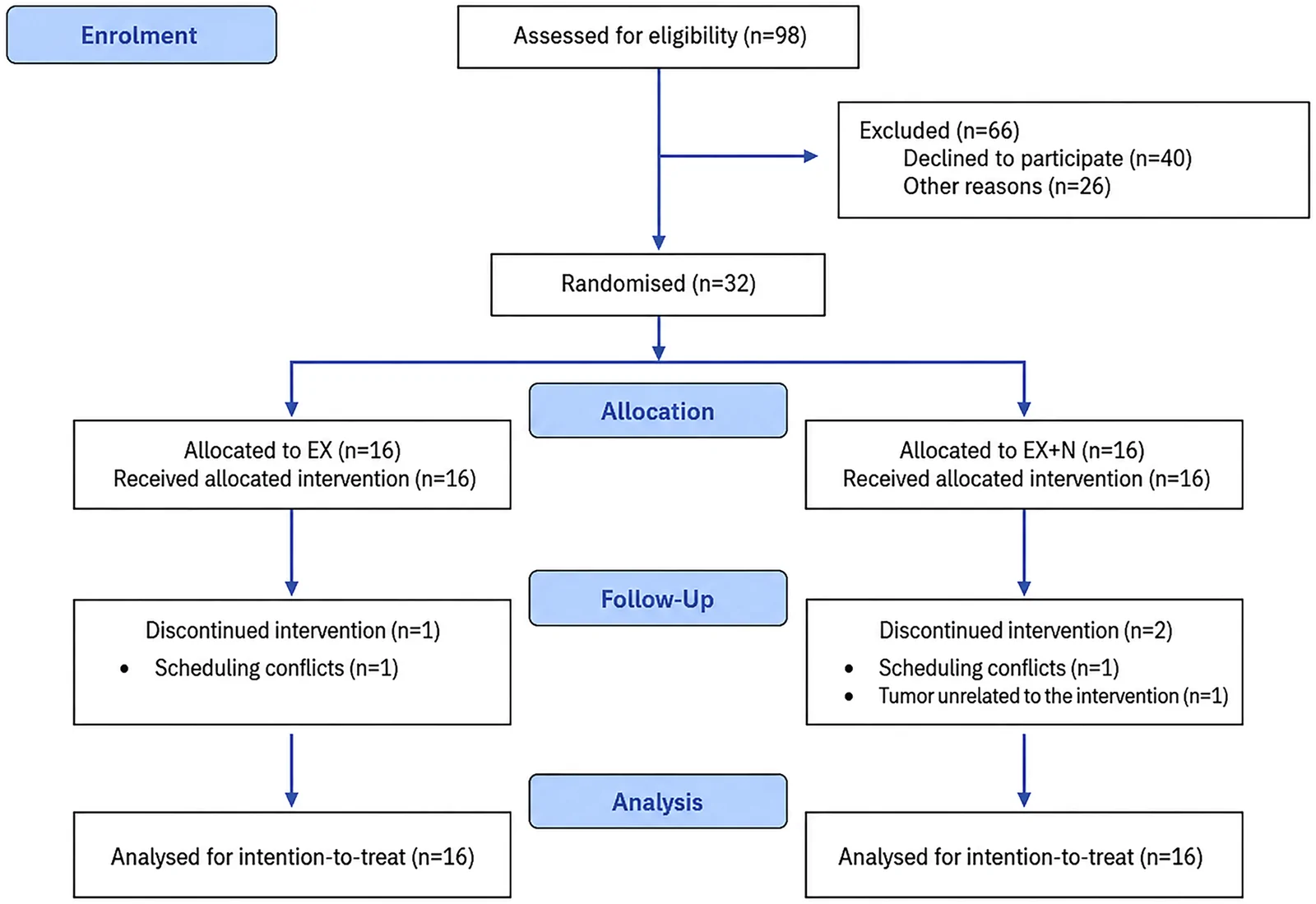
Flow diagram of participant allocation in the pragmatic randomized controlled trial. Participants were assigned to combined exercise training (EX; n = 16) or combined exercise training plus nutritional counseling (EX + N; n = 16) over 12 weeks.

A total of 32 participants were randomized, and 29 completed the study (Fig. 1). Three participants discontinued the intervention and were retained in the intention-to-treat analysis: one in EX and one in EX + N because of scheduling conflicts, and one in EX + N because of a tumor diagnosis unrelated to the intervention. There were no significant differences in the baseline characteristics between groups (Table 1). No major adverse events or complications were observed during the study. The attendance rate was 75 ± 15% (18 sessions) in EX and 83 ± 12% (20 sessions) in the EX + N group, with no difference between groups (*P* = 0.148). Attendance at the nutritional counseling sessions was 100% in the EX + N group.

**Table 1:** Baseline demographic and clinical characteristics of the participants.

Variables	EX (n = 16)	EX + N (n = 16)	*P* value
Age, years	62 ± 7	62 ± 5	0.833
**Anthropometry**			
Body weight (kg)	79 ± 16	79 ± 10	0.997
Height (cm)	166 ± 1	168 ± 1	0.872
BMI (kg/m²)	28 ± 4	28 ± 3	0.757
Waist circumference (cm)	100 ± 11	97 ± 8	0.413
**Sex, n(%)**			
Men	10 (63)	9 (56)	1000
Women	6 (38)	7 (44)	
**Etnicity, n(%)**			
White	9 (56)	14 (88)	0.113
Black	7 (44)	2 (13)	
**Glycemic status (fasting glucose)**			
Normal (<100 mg/dL)	11 (69)	8 (50)	0.613
Prediabetes (100–125 mg/dL)	3 (19)	5 (31)	
Diabetes (≥126 mg/dL)	2 (13)	3 (19)	
**Medications, n(%)**			
Diuretics	6 (38)	7 (44)	0.513
β blockers	2 (13)	4 (25)	0.325
ARBs	9 (56)	7 (44)	0.598
CCB	4 (25)	3 (19)	1000
ACEI	7 (44)	6 (38)	0.829
Diabetes	0 (0)	3 (18)	0.062

Values are mean ± SD or n (%).

*EX*, combined exercise training alone; *EX + N*, combined exercise training plus nutritional counseling; *BMI*, body mass index; *ARBs*, angiotensin II receptor blockers; *CCB*, calcium channel blockers; *ACEI*, angiotensin-converting enzyme inhibitors.

Bold *P*-values indicate significant results (P < 0.05).

**Fig. 2:**
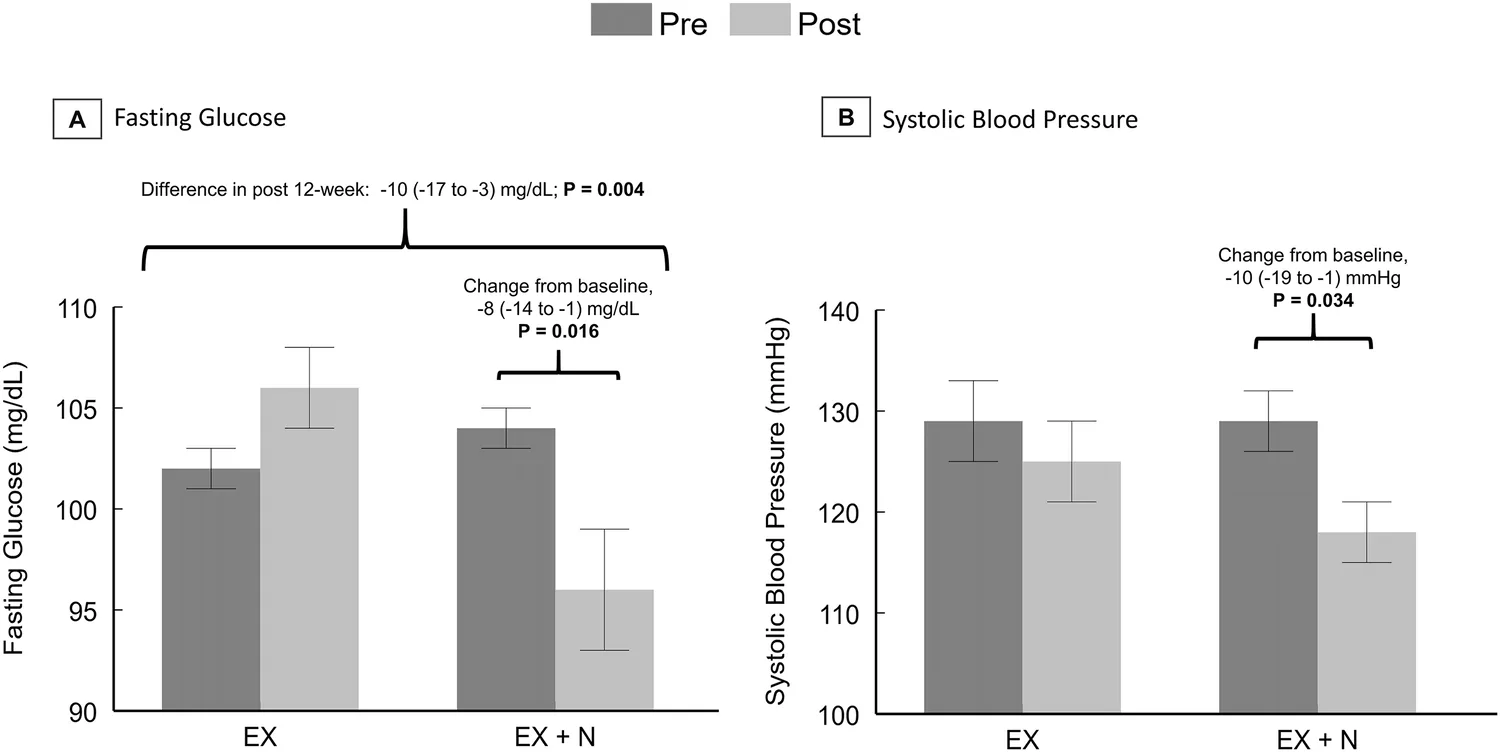
Changes in metabolic and cardiovascular markers pre- and post-intervention. Fasting blood glucose (**A**) and systolic blood pressure (**B**) at baseline (Pre) and post-intervention (Post) in combined training alone (EX) and combined exercise training plus nutritional counseling (EX + N) groups. Data are presented as mean ± standard error.

Fasting blood glucose decreased in the EX + N group (*P* = 0.016; g = 1.00), whereas it increased in the EX group (*P* = 0.040; g = 0.63). The between-group difference in post-intervention values favored the EX + N group (*P* = 0.004; g = 1.07) (Fig. 2A).

Office systolic blood pressure decreased in the EX + N group (*P* = 0.034; g = 0.89), with no significant change in the EX group (*P* = 0.287; g = 0.19). The between-group difference was not significant (*P* = 0.220; g = 0.43) (Fig. 2B). No significant pre–post changes or between-group differences were observed in diastolic blood pressure (*P* ≥ 0.05). Within the EX + N group, pulse pressure decreased (*P* = 0.042; g = 0.78), with no significant change in the EX group and no between-group difference. No significant within- or between-group changes were observed for diastolic blood pressure, mean arterial pressure, heart rate, double product, body composition, or cardiorespiratory fitness (*P* ≥ 0.05). Within- and between-group estimates with the corresponding effect sizes for all outcomes are reported in Table 2, whereas Fig. 2 illustrates the outcomes with significant changes.

**Table 2:** Cardiovascular risk outcomes at baseline and after the 12-week combined training intervention, with or without nutritional counseling.

Variables	EX (*n* = 16)					EX + N (n = 16)					Between-Group difference in final		
	Baseline	Final	∆	*P* value	Effect size	Baseline	Final	∆	*P* value	Effect size		*P* value	Effect size
Fasting glucose (mg/dL)	102 ± 1	106 ± 2	4 (1 to 7)	**0.040**	0.63	104 ± 1	96 ± 3	−8 (−14 to −1)	**0.016**	1.00	−10 (−17 to −3)	**0.004**	1.07
**Hemodynamic measurements**													
SBP	129 ± 4	125 ± 4	−3 (−9 to 2)	0.287	0.19	129 ± 3	119 ± 3	−10 (−19 to −1)	**0.034**	0.89	−6 (−17 to 4)	0.220	0.43
DBP	79 ± 3	79 ± 3	0 (−3 to 4)	0.908	0.00	80 ± 2	75 ± 2	−4 (−9 to 1)	0.086	0.56	−3 (−10 to 4)	0.376	0.44
MAP	97 ± 3	99 ± 3	2 (−2 to 6)	0.362	0.13	98 ± 2	95 ± 2	−2 (−9 to 3)	0.403	0.30	−3 (−12 to 4)	0.370	0.36
HR	70 ± 3	67 ± 2	−3 (−7 to 1)	0.087	0.24	67 ± 2	70 ± 3	2 (−1 to 6)	0.221	0.25	3 (−4 to 12)	0.407	0.71
DP	9076 ± 505	8365 ± 385	−710 (−1449 to 27)	0.059	0.36	8784 ± 387	8383 ± 533	−401 (−1351 to 549)	0.408	0.21	18 (−1271 to 1306)	0.979	0.17
PP	50 ± 3	46 ± 2	−3 (−7 to 1)	0.066	0.33	49 ± 2	42 ± 2	−6 (−12 to −1)	**0.042**	0.78	−3 (−10 to 3)	0.312	0.36
**Anthropometry measurements**													
Body weight (kg)	79 ± 3	80 ± 3	1 (−1 to 1)	0.328	0.08	79 ± 2	79 ± 2	0 (−1 to 1)	0.861	0.00	−1 (−9 to 8)	0.914	0.09
BMI (kg/m²)	28 ± 1	28 ± 1	0 (−1 to 1)	0.220	0.00	28 ± 1	28 ± 1	0 (−1 to 1)	0.947	0.00	−0 (−3 to 1)	0.603	0.00
Waist circumference (cm)	100 ± 2	98 ± 2	−2 (−3 to 1)	0.100	0.48	96 ± 2	96 ± 2	0 (−3 to 2)	0.589	0.00	−2 (−9 to 3)	0.384	0.30
**Six-Minute Walk Test**													
VO₂max (mL·kg⁻¹·min⁻¹)	29 ± 1	29 ± 1	0 (−1 to 1)	0.855	0.00	28 ± 1	26 ± 1	−2 (−3 to 1)	0.092	0.48	−3 (−6 to 1)	0.144	0.73
Distance (m)	571 ± 19	571 ± 20	0 (−34 to 34)	0.955	0.00	613 ± 20	572 ± 21	−41 (−85 to 3)	0.150	0.48	1 (−57 to 59)	0.974	0.01

Values are mean ± SE or mean (95% CI). Bold p-values indicate significant results (p < 0.05). Negative or positive differences in the between-group difference in final are in favor of the EX + N group.

*SBP*, systolic blood pressure (mmHg), *DBP*, diastolic blood pressure (mmHg), *MAP*, mean arterial pressure (mmHg), *HR*, heart rate (bpm); *DP*, rate-pressure product (mmHg.bpm), *PP*,pulse pressure (mmHg), VO₂max Maximal oxygen uptake.

## Discussion

The main result of this pragmatic trial demonstrates that the integration of nutritional counseling into a community-based exercise program significantly improves glycemic control. The within-group reduction in systolic blood pressure in EX + N but not in EX also suggests a favorable cardiovascular benefit when using a combined approach. These benefits were achieved with twice-weekly exercise and bi-weekly nutritional sessions, underscoring the clinical relevance of this multidisciplinary approach.

The reduction observed in the EX + N group on fasting blood glucose (≈8 mg/dL) occurred in participants who had a mean baseline fasting glucose in the prediabetes range, a range associated with increased risk of type 2 diabetes and cardiovascular disease [26]. Although this magnitude is smaller than the glycemic shift typically associated with a 1% reduction in glycated hemoglobin24 (≈24 mg/dL), the between-group difference of 10 mg/dL is clinically relevant, since modest reductions within the prediabetes range can mitigate cardiovascular risk [27, 28]. The use of a pragmatic nutritional counseling intervention, delivered without energy restriction, differed from intensive weight-loss interventions [7, 29]. Although dietary intake was not objectively quantified, the glycemic improvement is likely attributable to enhanced diet quality (increased fiber, reduced ultra-processed food consumption, among others), which improves insulin sensitivity independent of significant weight loss [30, 31]. In contrast, EX group did not modify fasting glucose levels. The absence of progression in exercise volume or intensity during the present exercise intervention limited additional metabolic adaptations, consistent with the dose-dependent nature of progressive exercise protocols [32]. These findings suggest that nutritional counseling effectively optimizes glycemic control when exercise exposure remains constant.

Despite a significant intragroup reduction in systolic blood pressure of approximately 10 mmHg within the EX + N group, no significant between-group differences emerged. The systolic blood pressure reduction observed within the EX + N group is clinically relevant, since each 5 mmHg reduction is associated with about a 9% lower risk of major cardiovascular events, which corresponds to roughly a 17% lower risk for the 10 mmHg reduction observed here [33]. The low baseline systolic blood pressure values (≈129 mmHg) may explain this outcome, as in individuals with well-controlled hypertension, additional reductions are often modest given that the magnitude of change depends largely on pre-intervention levels [34]. Although exercise training alone is well established for blood pressure management [35], the absence of progressive overload, combined with a low weekly exercise frequency [36], may have limited additional hemodynamic adaptations in the EX group. It is also important this reduction occurred within the EX + N group only, with no significant between-group difference, and the study was not powered to detect blood pressure changes, so this interpretation should be viewed as exploratory. Future trials with larger samples are necessary to confirm the observed trend of superiority translates into statistically significant clinical advantage.

Physical fitness parameters remained stable throughout the study. Participants presented as a previously trained cohort with estimated VO_₂max_ levels above age-adjusted reference values, suggesting a ceiling effect for functional improvements [22]. Cardiorespiratory fitness gains are typically smaller in healthy older adults than in individuals with functional limitations [37], and the absence of exercise progression in this community program likely limited further aerobic adaptations. Thus, preserving functional capacity over 12 weeks in this already-trained population likely reflects physiological stability rather than lack of intervention efficacy [38].

The nutritional counseling prioritized qualitative dietary patterns and the avoidance of ultra-processed foods [6, 14], which could help to explain why anthropometric markers remained unchanged, as the intervention targeted nutrient density rather than caloric deficit. The improvements in insulin sensitivity and glycemic control can occur independent of major weight loss through mechanisms such as increased fiber intake and reduced systemic inflammation [30, 31]. Furthermore, this weight-neutral metabolic improvement is particularly relevant for older adults, as it prioritizes glycemic health while avoiding the risks of muscle mass loss often associated with restrictive diets [39].

A main strength of this study is its pragmatic and accessible intervention model. The program combined nutritional counseling with bodyweight exercises that require no specialized equipment or facilities and can be performed at home or in public and community spaces. This low-cost framework reduces barriers to access and enhances external validity and implementation feasibility [40, 41]. Unlike intensive laboratory-controlled interventions [29], this model prioritizes long-term sustainability and adherence, and it produces changes in fasting blood glucose even among participants with prior training experience [42]. Because it relies on strategies that fit everyday routines, the intervention is plausible to sustain within primary care and community health programs, where supervised high-cost interventions are rarely feasible [43].

This study has some limitations. The trial was not registered in a public clinical trials registry, which limits the independent verification of the pre-specified outcomes and analysis plan. The sample size, while powered for fasting glucose, may have been insufficient to detect between-group differences in hemodynamic parameters. Glycemic control was assessed via fasting glucose rather than glycated hemoglobin, and the use of capillary measurements may introduce greater variability than laboratory assays. The exercise load was not progressed during the program, which may have limited the magnitude of the adaptations, particularly for cardiorespiratory fitness and blood pressure. Additionally, the lack of objective dietary quantification limits mechanistic inferences regarding specific nutrient changes. Baseline dietary knowledge was not formally assessed, which limits interpretation of the extent to which the counseling changed pre-existing knowledge. Despite these constraints, the study’s strengths include a randomized controlled design with blinded outcome assessment and an intention-to-treat analysis. Furthermore, the community-based, low-cost framework enhances external validity and implementation feasibility. Unlike intensive laboratory-controlled interventions, this pragmatic model prioritizes long-term sustainability and adherence, proving effective even for participants with prior training experience who had already stabilized their results.

In older adults with hypertension, adding nutritional counseling to a community-based combined exercise program reduced fasting blood glucose compared with exercise alone. Blood pressure decreased within the group receiving nutritional counseling, but the absence of between-group differences and the limited statistical power for blood pressure mean these effects should be interpreted with caution. The pragmatic, low-cost, and community-based approach of the intervention supports its applicability in real-world settings.

## SUMMARY

### What is known about this topic

- Combined exercise training is a cornerstone non-pharmacological strategy for managing blood pressure and glucose levels in older adults.
- Nutritional interventions focusing on dietary patterns independently improve cardiovascular outcomes.
- Current evidence on combined exercise and nutrition often stems from controlled trials, which may lack the external validity and adherence seen in community settings.

### What this study adds

- This pragmatic trial demonstrates that adding low-frequency nutritional counseling (biweekly) to a community exercise program significantly reduces fasting glucose compared to exercise alone.
- The combined approach was effective even in previously active older adults, suggesting that nutritional guidance can help overcome physiological plateaus in metabolic health.

## Supplementary information

Supplementary material

## Data Availability

The datasets generated during and/or analyzed during the current study are available from the corresponding author on reasonable request.
