# Supplementary material for: Effects of combined exercise training with or without nutritional counseling on cardiovascular risk factors in older adults with hypertension: a pragmatic randomized trial
Source: J Hum Hypertens. 2026 Sep 3;40(11):880–7. doi: 10.1038/s41371-026-01217-7 (PMC13747805; doi:10.1038/s41371-026-01217-7)
Supplement: Supplementary file 1 [file 41371_2026_1217_MOESM1_ESM.docx]

**Supplementary Figure 1.** PRECIS-2 wheel illustrating the degree of pragmatism of the trial across the nine design domains (eligibility, recruitment, setting, organisation, flexibility of delivery, flexibility of adherence, follow-up, primary outcome, and primary analysis). Each domain was rated from 1 (very explanatory) to 5 (very pragmatic).
